# Green Line on the Gum from Copper Poisoning

**Published:** 1870-02

**Authors:** 


Green Line on the Gum from Copper Poisoning.—By Donald Fraser, M. D., Montreal.—Trusting that the following may not be without interest to your readers, I crave indulgence for a small space of your valuable journal, for its insertion. Some six or seven months ago, I was kindly shown by Dr. Gervis, Assistant Physician to the St. Thomas' Hospital, London, England, a patient suffering from chronic poisoning by copper. *A green line on the gum*, analagous to the blue line seen in cases of poisoning by lead, was distinctly visible. The patient was a sailor just returned from a long voyage, and had received the poison through the medium of the lime juice which had been kept in a copper vessel. This was the second case of the kind which had come under the notice of this gentleman, who is, I believe, the first to notice the fact.—*Canada Journal of Dental Science.*

